# Quadrotor with wheels: design and experimental evaluation

**DOI:** 10.1038/s41598-024-66396-0

**Published:** 2024-07-06

**Authors:** Ilan Aizelman, Dan Magazinnik, Dan Feldman, Itzik Klein

**Affiliations:** 1 The Hatter Department of Marine Technologies, University of Haifa, 199 Aba Khoushy Ave., 3103301 Mount Carmel, Israel; 2 Department of Computer Science, University of Haifa, 199 Aba Khoushy Ave., 3103301 Mount Carmel, Israel

**Keywords:** Engineering, Mechanical engineering

## Abstract

Quadrotors have found widespread use in indoor applications, including tracking and mapping. In general, to carry out such tasks effectively, a navigation solution should provide both accuracy and battery efficiency. To achieve both, we propose a cost-effective and lightweight wheeled quadrotor that combines both driving and flying capabilities. Our design allows the quadrotor to perform both functions seamlessly. We provide a detailed description of the design and construction process, highlighting its advantages. Our focus was on the Tello quadrotor, which weighs 80 grams. Our design allowed driving capability with an increased weight of only fifteen grams, resulting in less than 20% of the added weight. Furthermore, we evaluate the quadrotor’s pure inertial navigation performance and corresponding battery consumption by employing various flying and driving patterns. Our results show that when only driving the battery consumption was the lowest with 10% and some flying scenarios improve the positioning error by more than 70%.

## Introduction

Quadrotor usage in indoor and outdoor applications has risen dramatically in the last decade, including applications in construction^1^, transportation^2^, surveillance^3^, maritime science^4^, and law enforcement^5^. In construction and industry, quadrotors are used to inspect construction, machines, or infrastructure. The utilization of quadrotors is not limited to outdoor applications. Indoors, quadrotors are used for manufacturing and structure inspection^6,7^, mapping^8^, and emergency response^9^. The inspection and monitoring of manufacturing sites, structures, and infrastructure are important issues for their sustainability and further maintenance. In the mapping field, quadrotors are utilized for the purposes of image recognition and mobile 3D mapping. This involves capturing images of specific regions with the quadrotor, which are then utilized to construct a virtual 3D model of the area. In emergency cases, quadrotors are used for rescue missions in buildings after natural disasters.

For localization and navigation, quadrotors can utilize a range of sensors, including cameras, inertial navigation system (INS)^10^, lidar^11^, or global navigation satellite systems (GNSS)^12,13^. Usually, outdoor applications heavily depend on GNSS signals to provide position and velocity information with accuracy suitable for various applications. As GNSS is not available indoors, other solutions exist to enable accurate navigation. One approach is using inertial sensors like accelerometers, gyroscopes, and magnetometers. With the inertial data, the pose of the quadrotor can be estimated. Since the inertial measurements contain error sources, the navigation solution drift is considerably fast^14^. Therefore, other sensors, such as a camera, can be fused to achieve a sufficiently accurate navigation solution. Most notably, cameras are used in simultaneous localization and mapping^15^ (SLAM) algorithms, whose goal is the construction and real-time updates of a map in an unknown environment while simultaneously keeping track of the platform’s location within it.

In the last decade, more emphasis has been placed on the topic of pluggable equipment for quadrotors, such as wheels or arms. There have been a few quadrotors constructed that can transition between driving and flying, such as the Flying STAR^16^, Flying Monkey^17^, MALV^18^, DALER^19^, HyTAQ^20^, climbing quadrotor^21,22^, and others^23,24^. Using the same motors, the Flying STAR can run and fly thanks to its sprawling mechanism and propellers. Although it can overcome larger obstacles, its body and legs contribute to its maneuverability limitations, the size of obstacles it can overcome is proportional to its body and leg sizes. The Flying Monkey features a foldable crawling mechanism located on the underside of a quadrotor, which enables the quadrotor to crawl when deployed. The MALV is a quadrotor with wings and wheels that aims to extend the versatility and adaptability of robots in complex environments. Furthermore, the fixed-wing quadrotor DALER can move on the ground by rotating its wings, and in order to assist in wall climbing, the quadrotor has four wheels coupled with motors tilted at an angle of about 45 degrees. The HyTAQ quadrotor has an additional configuration, it consists of a quadrotor hinged inside a cage, which reduces disturbances upon collision with external obstacles and serves as a wheel for ground locomotion.

Driving capabilities for quadrotors are useful for several situations, such as flight restrictions due to no-fly zones, bad weather, or heavy air traffic. Although the designs of^16,20–22^ are sophisticated, their overall weight is almost one kilogram and they require a tremendous amount of energy to drive. For indoor applications, driving capabilities will assist in maneuvering through small spaces and enable lower battery consumption which allowing more time to complete a mission.

In this paper, we propose and design a low-cost and low-weight wheeled quadrotor for indoor applications. To that end, we employ the DJI Tello^25^ quadrotor due to its low weight, onboard sensors, potential for rapid prototyping, and quick design iterations. By adding the electro-mechanical components that enable driving capabilities, we achieve a simple, lightweight, cheap, and efficient hybrid quadrotor.

The primary contribution of the research is the development of a cost-effective and lightweight wheeled quadrotor for indoor applications, which also serves as a versatile research platform. Our design sets itself apart from other systems in terms of weight, complexity, degrees of freedom, activation model, and available sensors. The wheeled quadrotor operates indoors with a navigation system containing a camera and inertial sensors. In some situations, poor lighting conditions may occur limiting the camera operation. As a result, the quadrotor navigation solely depends on inertial sensors. As their measurements contains noise and other error terms, the navigation solution drifts. To evaluate the navigation solution drift and examine means to mitigate it, an empirical analysis is conducted. We examine different dynamics for pure inertial navigation and quantify the performance in terms of accuracy and battery consumption.

There are a number of practical applications for our hybrid quadrotor, including:

1. **Deliveries**: The quadrotor can be employed to transport small items, such as medicine, from one location to another. Its compact size and maneuverability make it suitable for navigating through tight spaces and reaching remote areas efficiently.
2. **Surveillance**: The quadrotor’s advantageous features make it an ideal choice for indoor surveillance. With its low battery consumption and minimal noise production during driving operation, it can discreetly monitor and gather information without causing disturbance or attracting attention.
3. **Mapping**: The integration of the SLAM algorithm enables the quadrotor to generate detailed maps of buildings. These maps can serve various purposes, such as architectural planning, navigation assistance, or even disaster management. The quadrotor’s ability to capture accurate and real-time data contributes to the effectiveness and reliability of the mapping process.

The rest of the paper is organized as follows: “Problem formulation” section gives inertial algorithms for determining the quadrotor pose in pure inertial navigation. In “Wheeled quadrotor design” section, the wheeled quadrotor design is presented, along with design motivation, the mechanical design of our quadrotor, and software design. Section “Experimental setup and design4” gives the experimental setup and flow, while “Analysis and results” section describes our analysis and results. Finally, “Conclusions and future work” section reports the conclusions of this study.

## Problem formulation

In this section, we present inertial algorithms to provide the pose of the quadrotor in pure inertial navigation.

### INS equations of motion

Two reference frames are addressed in this work: the inertial frame denoted as i-frame and the body frame denoted as b-frame. The i-frame is located at the quadrotors starting point while the b-frame coincides with the inertial sensors’ sensitive axes.

Given initial conditions and inertial sensor readings, the INS equations provide the navigation solution consisting of the position, velocity, and origination of the platform. The specific force vector is measured by the accelerometers and is denoted as:

1 $$\begin{aligned} {\varvec{f}}_{ib}^{b}= \begin{bmatrix} f_x \\ f_y \\ f_z \end{bmatrix} \end{aligned}$$

where ${\varvec{b}}$ denotes body frame and ${\varvec{i}}$ denotes inertial frame. Additionally, the angular velocity vector is measured by the gyroscopes and denoted as:

2 $$\begin{aligned} {\varvec{\omega }}_{ib}^{b}= \begin{bmatrix} {\omega }_x \\ {\omega }_y \\ {\omega }_z \end{bmatrix} \end{aligned}$$

where ${\omega }_x$, ${\omega }_y$, and ${\omega }_z$ are the vector components of the angular velocity vector as measured along the gyroscopes’ sensitive axes.

The angular velocity vector and specific force vector, combined with the initial conditions, are used to solve the INS equations of motion to obtain the navigation solution: position, velocity, and attitude. The equations that are used in order to estimate the motion of the quadrotor^26^ are given by:

3 $$\begin{aligned} {\varvec{\dot{p}}}^{n}= & {} {\varvec{v}}^{n} \end{aligned}$$

4 $$\begin{aligned} {\varvec{\dot{v}}}^{n}= & {} {\varvec{T}}^{n}_{b}{\varvec{f}}_{ib}^{b} + {\varvec{g}}^{n}\end{aligned}$$

5 $$\begin{aligned} {\varvec{\dot{T}}}^{n}_{b}= & {} {\varvec{T}}^{n}_{b}{\varvec{\Omega }}_{ib}^{b} \end{aligned}$$

where ${\varvec{p}}^{n}$ is the position vector expressed in the local navigation frame, ${\varvec{v}}^{n}$ is the velocity vector expressed in the navigation frame, ${\varvec{g}}^{n}$ is the local gravity vector expressed in the navigation frame, ${\varvec{\Omega }}_{ib}^{b}$ is a skew-symmetric form of the angular velocity vector ${\varvec{\omega }}_{ib}^{b}$ and is defined by:

6 $$\begin{aligned} {\varvec{\Omega }}_{ib}^{b}=\begin{bmatrix}0&{} -{\omega }_z&{}{\omega }_y\\ {\omega }_z&{}0&{}-{\omega }_x\\ -{\omega }_y&{}{\omega }_x&{}0\end{bmatrix} \end{aligned}$$

${\varvec{T}}^{n}_{b}$ is the transformation matrix from the body frame to the navigation frame and is defined by^27^:

7 $$\begin{aligned} {\varvec{T}}^{n}_{b}=\begin{bmatrix}c_{\theta }c_{\psi }&{}s_{\phi }s_{\theta }c_{\psi }-c_{\phi }s_{\psi }&{}c_{\phi }s_{\theta }c_{\psi }+s_{\phi }s_{\psi }\\ c_{\theta }s_{\psi }&{}s_{\phi }s_{\theta }s_{\psi }+c_{\theta }c_{\psi }&{}c_{\phi }s_{\theta }s_{\psi }-s_{\phi }c_{\psi }\\ -s_{\theta }&{}s_{\phi }c_{\theta }&{}c_{\phi }c_{\theta }\end{bmatrix} \end{aligned}$$

where $s_{x}$ is the sine of *x*, $c_{x}$ is the cosine of *x*, $\phi$, $\theta$ and $\psi$ are the roll, pitch, and yaw angles respectively.

### Two dimensional INS

A special case in the INS equations appears when the quadrotor drives on a flat surface, assuming the roll and pitch angles can be neglected. In that case, the accelerometer measurements on the z-axis are nullified. Hence, the specific force vector is now:

8 $$\begin{aligned} {\varvec{f}}_{ib {2D}}^{b}= \begin{bmatrix} f_x \\ f_y \\ 0 \end{bmatrix} \end{aligned}$$

Due to the same assumption, the angular velocity vector is:

9 $$\begin{aligned} {\varvec{\omega }}_{ib {2D}}^{b}= \begin{bmatrix} 0 \\ 0 \\ {\omega }_z \end{bmatrix} \end{aligned}$$

Thus, (6) reduces to:

10 $$\begin{aligned} {\varvec{\Omega }}_{ib {2D}}^{b}=\begin{bmatrix}0&{} -{\omega }_z&{}0\\ {\omega }_z&{}0&{}0\\ 0&{}0&{}0\end{bmatrix} \end{aligned}$$

and (7) to:

11 $$\begin{aligned} {\varvec{T}}^{n}_{b {2D}}=\begin{bmatrix}c_{\psi }&{}-s_{\psi }&{}0\\ s_{\psi }&{}c_{\psi }&{}0\\ 0&{}0&{}1\end{bmatrix} \end{aligned}$$

### Inertial measurement unit (IMU): first order calibration

The most basic calibration procedure for an IMU involves determining the offset for each axis of both the gyroscope and accelerometer. The coefficients can be determined by initially collecting readings while it is assumed that the IMU is stationary and subsequently utilizing these values as reference points when obtaining measurements in future instances. Instead of using higher-order calibration routines, this calibration method represents a basic approach for the IMU and is generally sufficient. The coefficients are denoted as:

12 $$\begin{aligned} {\varvec{b}}_{\omega }= \begin{bmatrix} {\Sigma }({\omega _{x1},..., \omega _{xn}}) / n\\ {\Sigma }({\omega _{y1},..., \omega _{yn}}) / n \\ {\Sigma }({\omega _{z1},..., \omega _{zn}}) / n \end{bmatrix} \end{aligned}$$

13 $$\begin{aligned} {\varvec{b}}_{f}= \begin{bmatrix} {\Sigma }({f_{x1},..., f_{xn}}) / n\\ {\Sigma }({f_{y1},..., f_{yn}}) / n \\ {\Sigma }({f_{z1},..., f_{zn}}) / n + g \end{bmatrix} \end{aligned}$$

where *n* denotes the number of samples measured. Notice that we apply first-order calibration to the accelerometers as we assume almost zero roll and pitch angles, resulting in the alignment of the body z-axis with the gravity direction.

### Quadrotor dead reckoning

**Figure 1:**
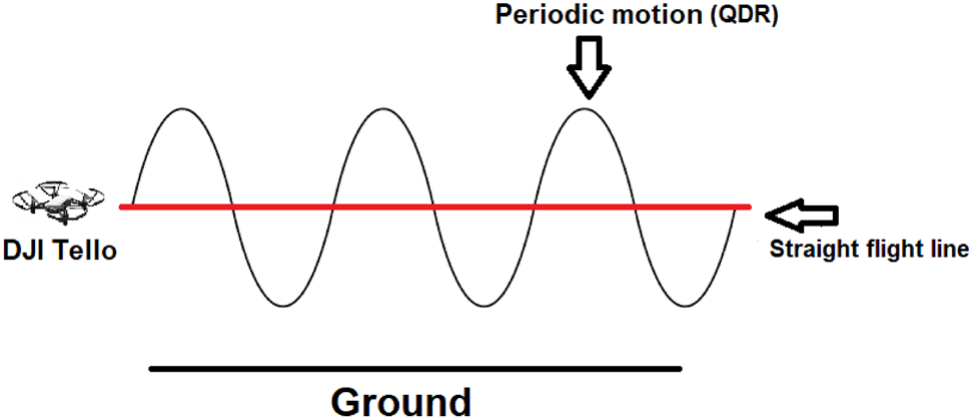
Periodic motion in QDR. This approach improves the pure inertial navigation solution.

The fundamental difficulty in pure inertial navigation scenarios is reducing the drift in the navigational solution brought on by the noisy measurements of the inertial sensors. To mitigate such unwanted behavior in the quadrotor dead reckoning (QDR) framework, instead of flying in a straight path, QDR requires the quadrotor to follow a periodic motion trajectory as shown in Fig. 1, and apply a dedicated algorithm.

The QDR process has two parts, which are very similar to the pedestrian dead reckoning (PDR)^28,29^ approach. Peak detection comes first, followed by a peak-to-peak distance estimate. Peak-to-peak distance may be computed using the Weinberg method^30^ once the peaks have been identified. The peak-to-peak distance is estimated by:

14 $$\begin{aligned} {d}_{w}= & {} {\varvec{G}}_{w}(max({\varvec{f}}^{b}_{p2p}) - min({\varvec{f}}^{b}_{p2p}))^{1/4} \end{aligned}$$

where ${\varvec{f}}^{b}_{p2p}$ is the set of all specific force magnitudes in the peak-to-peak duration, and the ${\varvec{G}}_{w}$ is the Weinberg gain. Before application of the QDR approach, the Weinberg gain is determined. This is done by flying the quadrotor in the expected dynamics for a known distance. Then, by finding the peak-to-peak intervals with their corresponding accelerometer readings, we sum the distances in (14) to the known travel distance and determine the Weinberg gain.

Using (5), the quadrotor’s attitude is determined in the same way as in conventional INS. For the horizontal QDR, instead of using (3), the quadrotor’s horizontal position is given by:

15 $$\begin{aligned} {\varvec{x}}_{k+1}= & {} {\varvec{x}}_{k}+{\varvec{d}}_{k}{\varvec{cos}}{\varvec{\psi }}_k \end{aligned}$$

16 $$\begin{aligned} {\varvec{y}}_{k+1}= & {} {\varvec{y}}_{k}+{\varvec{d}}_{k}{\varvec{sin}}{\varvec{\psi }}_k \end{aligned}$$

where ${\varvec{d}}_{k}$ is the peak-to-peak distance estimated using the Weinberg approach.

The same principles apply to the vertical QDR but instead of using (15)–(16) the following propagation equations are used:

17 $$\begin{aligned} {\varvec{x}}_{k+1}= & {} {\varvec{x}}_{k}+{\varvec{d}}_{k}{\varvec{cos}}{\varvec{\theta }}_k \end{aligned}$$

18 $$\begin{aligned} {\varvec{z}}_{k+1}= & {} {\varvec{z}}_{k}+{\varvec{d}}_{k}{\varvec{sin}}{\varvec{\theta }}_k \end{aligned}$$

where $\theta$ is the pitch angle.

Notice that the above equations for horizontal and vertical motion assume zero roll and pitch for the horizontal dynamics and constant yaw for the vertical motion.

The model-based QDR technique provides a solution that has several shortcomings:

- Pre-calibration is necessary for the Weinberg gain in (14), which is quite sensitive to the kind of periodic motion used in quadrotors.
- The quadrotor’s altitude is not provided by the QDR technique.
- QDR only offers a position solution (p2p) between two subsequent peaks, with a time interval of several hundred milliseconds between the two peaks.

To overcome those shortcomings, deep-learning QDR approaches were suggested^31^ yet they are not the main topic of this paper.

## Wheeled quadrotor design

### Design motivation

The solutions referenced in the works of^16–22,32,33^ facilitated quadrotor driving or walking abilities. However, these solutions necessitate the incorporation of numerous hardware components, thereby increasing the weight and complexity of the system. The primary aim of our wheeled quadrotor design is to attain driving capabilities with minimal addition of lightweight components.

The DJI Tello has been selected as a research platform to enhance its out of the box capabilities through various means .The quadrotor’s lightweight nature, onboard sensors, and potential for rapid prototyping and design iterations serve as the driving force behind our motivation to leverage these advantages in order to overcome inherent limitations of the DJI Tello, such as battery life, through the aforementioned methods.

### Mechanical design

There were several factors that influenced the mechanical design choices. Two main considerations revolved around lighting conditions in the environment. The secondary focus was on ensuring that pluggable equipment does not interfere with Tello’s built-in sensors. To achieve optimal weight and dimensions, we opted for lightweight gear motors made of plastic instead of metal. In addition, the 3D chassis was constructed with a material of high strength, minimizing its volume. Our design goals are targeted for indoor conditions.

We adopted the DJI Tello quadrotor due to its low weight and flexible design. To keep a low-weight quadrotor goal, the mechanical architecture of the quadrotor was kept as simplistic as possible. The DJI Tello has a variety of sensors and an onboard camera. It weighs eighty grams (propellers and battery included), has a down-facing range finder sensor, a barometer, 2.4 GHz 802.11n Wi-Fi, and 720p live video, among many other built-in features, as can be seen in Table 1. Additionally, the Tello platform exhibits excellent battery efficiency, when the built-in camera is turned off the battery lasts for one hour in driving-only mode, and around ten minutes in fly-only mode.

**Table 1:** DJI Tello technical specifications.

Quadrotor	Description
Weight	Approximately 80 g (propellers and battery included)
Dimensions	$98\times 92.5\times 41$ mm
Propeller	3 inches
Built-in functions	Range finder, barometer, LED, vision system, 2.4 GHz 802.11n Wi-Fi, 720p Live View
Port	Micro USB charging port
Flight performance max flight distance	100 m
Max speed	8 m/s
Max flight time	13 min
Max flight height	30 m
Battery detachable battery	1.1 Ah/3.8 V
Camera photo	5 MP ($2592\times 1936$)
FOV	$82.6^{\circ }$
Video	HD720P30
Format	JPG (photo), MP4 (video)

As part of our design, we attach to the bottom of the quadrotor a 3D-printed wheel chassis weighing 3.5 grams. Additionally, two tiny cylindrical gear motors are incorporated. They consist of a coreless brushed DC motor and a 136:1 plastic planetary gearbox. Each motor has a diameter of six millimeters and weighs 1.25 grams. For motor control, a motor module (DRV8835) is utilized. This tiny dual H-bridge motor driver weighs half a gram and enables bidirectional control of the two brushed DC motors.

**Figure 2:**
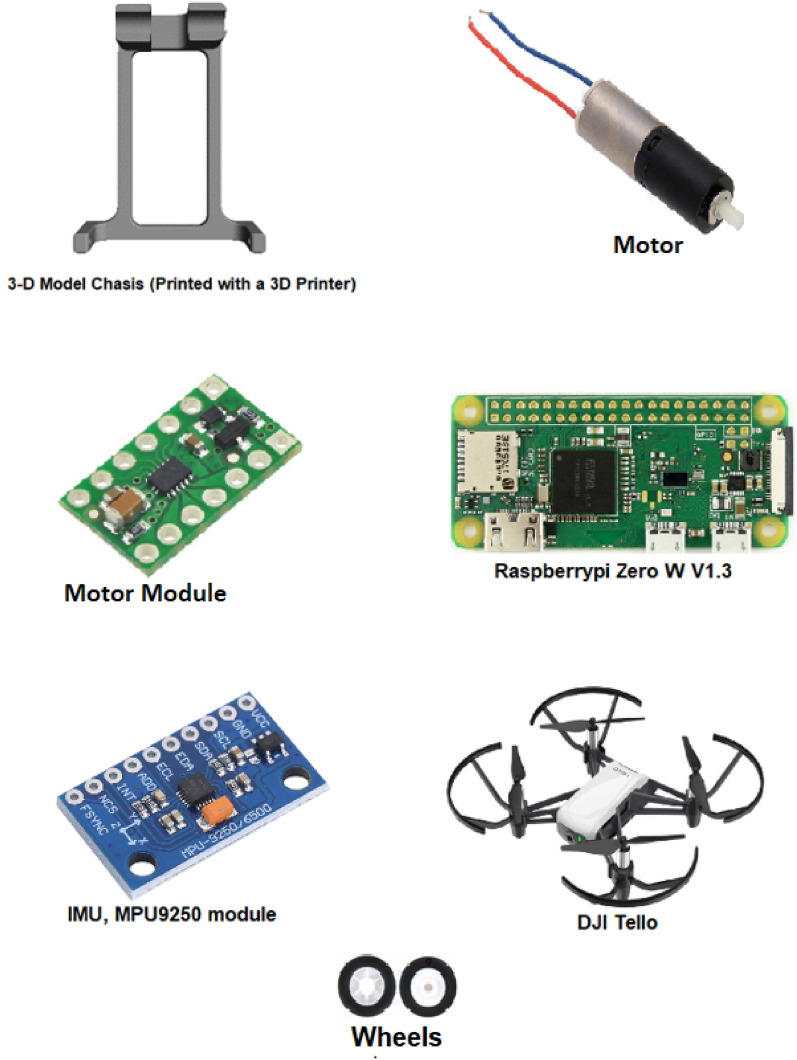
The system consists of a DJI Tello and six hardware components.

On top of the DJI Tello, an IMU (MPU9250) module is installed, weighing 2.7 grams. This module is a System in Package (SiP) that combines two chips: the MPU-6500, which houses a three-axis gyroscope, a three-axis accelerometer, and an onboard digital motion processor (DMP) capable of processing complex MotionFusion algorithms, and the AK8963 chip, which contains a three-axis digital compass. The IMU module is connected to a Raspberry Pi (RPi) Zero W, Version 1.3, as seen in Fig. 2 and is used as a light-weight computer for many purposes, such as:

- Recording different parameters, for example, the position of our wheeled quadrotor.
- Power on both the DRV8835 and MPU9250 modules.
- Quadrotor navigation through the experiment.

**Figure 3:**
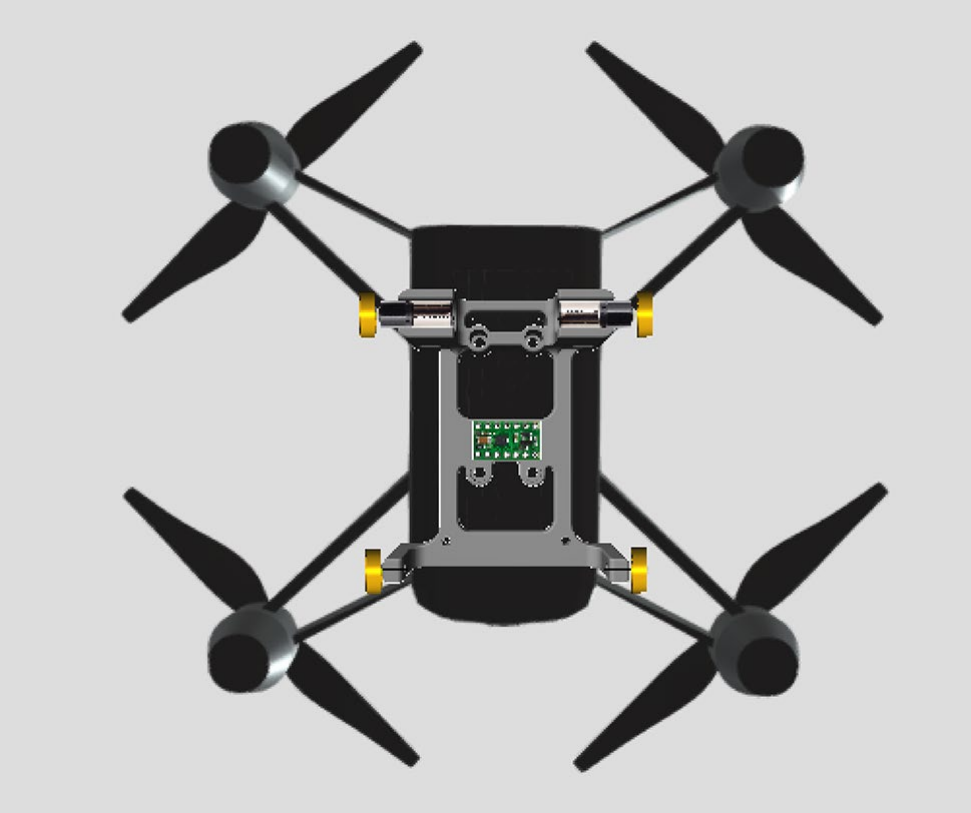
Our quadrotor bottom-view showing the driving configuration consisting of four wheels, two motors, motor module, 3D-printed chassis, and the DJI Tello.

**Figure 4:**
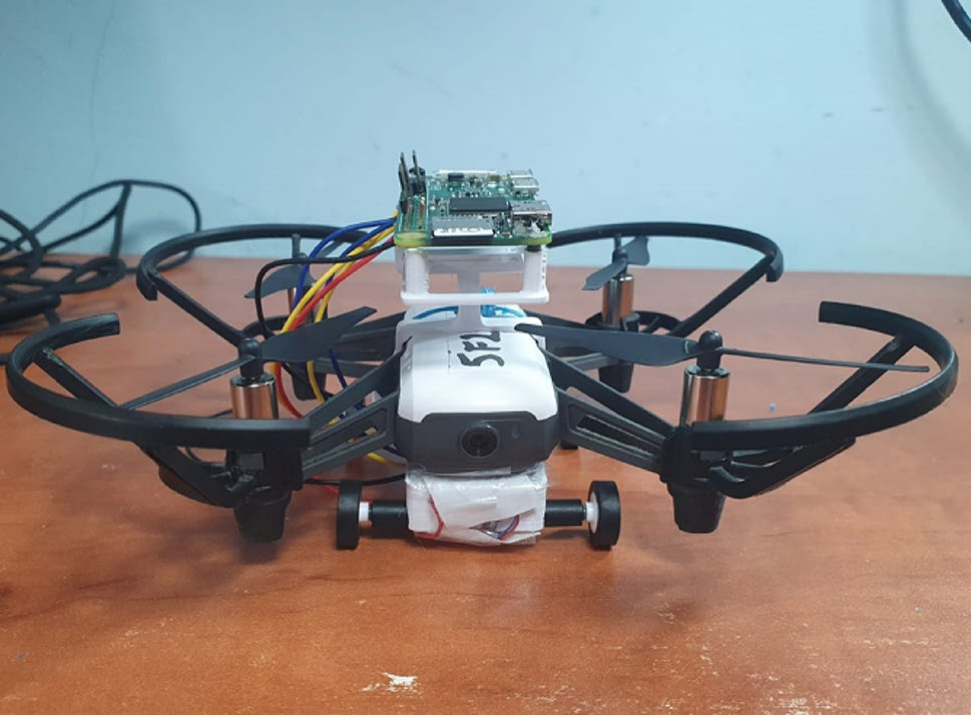
Our assembled hybrid quadrotor with driving and flying capabilities.

Figure 3 shows the quadrotor component assembly from the bottom. The total weight of our design is fifteen grams, resulting in less than 20% added weight. Our simple, lightweight, low-cost, and efficient hybrid quadrotor is shown in Fig. 4.

The mechanical design cost was approximately 45$ consisting the purchase and design of Raspberry Pi Zero W, sub-micro plastic planetary gear motor, DRV8835 dual motor driver carrier, micro-plastic wheels, MPU9250, 3D printed chassis, bolts, and other accessory equipment. If access to the quadrotor computer and sensors is available, the cost is reduced to approximately 20$.

### Software design

Two different design environments were used. The first is the robot operating system (ROS)^34^ which is an open-source framework that provides standard services such as hardware abstraction, low-level device control, implementation of commonly used functionality, message passing between processes, and package management. It is essential that ROS have a complete source code that is accessible to everyone. This is essential to making the entire software stack’s debugging process easier. This is especially true when hardware and several software tiers are being created and tested concurrently. Thus, it is an ideal environment for the SLAM algorithm. The second environment is the Raspberry Pi operating system (RPi OS)^35^, it runs Linux (a variety of distributions), and its primary supported operating system, Pi OS, is open-source and makes use of a number of different applications that are then utilized for our driving system and IMU recordings.

## Experimental setup and design

**Figure 5:**
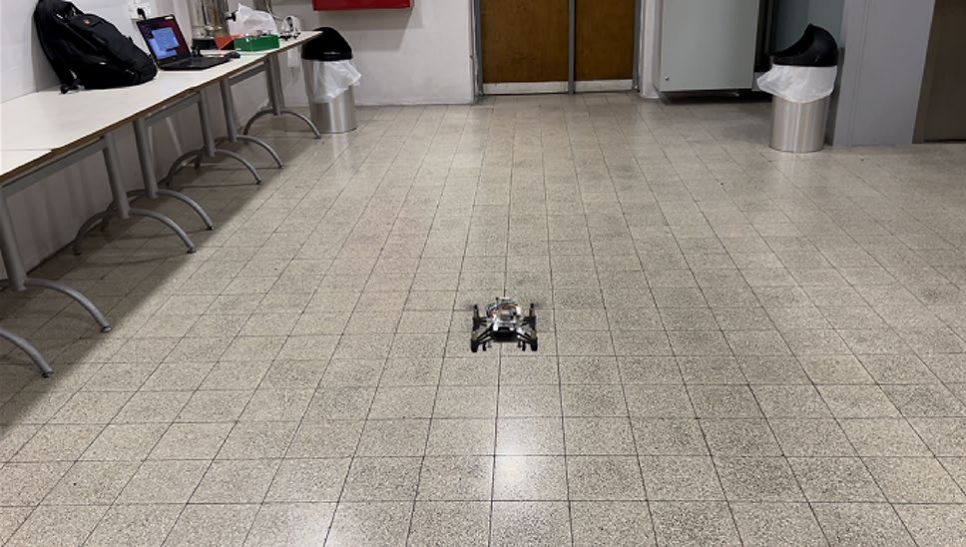
Our hybrid quadrotor in the experiment environment.

The experiments were carried out utilizing our modified DJI Tello with numerous sensors, a RPi, and a remote PC, as detailed in “Mechanical design” section. All of the experiments were conducted in a laboratory under comparable conditions: a square-shaped floor, no wind, and adequate illumination to locate features using SLAM and optical flow^36^. The experiment environment can be seen in Fig. 5.

Each experiment starts by turning on two components: the first is our hybrid quadrotor, which also turns on the Raspberry Pi automatically, and the second is the remote computer. The computer’s user interface (UI) is then activated, and the IMU is calibrated. Next, a first-order calibration, as described in “Inertial measurement unit (IMU): first order calibration” section, is applied for 10 seconds. This produces the calibration coefficients that are needed in order to obtain accurate readings from the three accelerometers and three gyroscopes while driving and flying. The influence of the inertial sensor calibration is presented in the next section.

The ORBSLAM2 algorithm^37^ is employed to provide a precise location at every point of the quadrotor trajectory. It was chosen because it does not require additional infrastructure, such as other available solutions: OptiTrack^38^, camera tracking^39^, radio frequency (RF)^40^, and terrestrial and satellite semantics tracking^41^. Moreover, the system works in real-time on a RPI in a wide variety of environments such as a laboratory. As a result, we are able to keep track of the quadrotor’s trajectory during each experiment until it arrives at its final eight-meter destination. The calibration process of SLAM involves executing a predetermined upward and downward movement of one meter. Upon completion of this motion by the quadrotor, the SLAM algorithm computes the coordinates, which are then utilized to determine the coefficients against the ground-truth data. The coefficients are denoted as:

19 $$\begin{aligned} \varvec{B}_{SLAM}= {H}_{SLAM} / 2 \end{aligned}$$

where $H_{SLAM}$ represents the vertical displacement achieved by the quadrotor. Simultaneously with the calibration process, the camera is turned on, and the SLAM algorithm tries to find good enough features^42^ to be successfully initialized.

**Figure 6:**
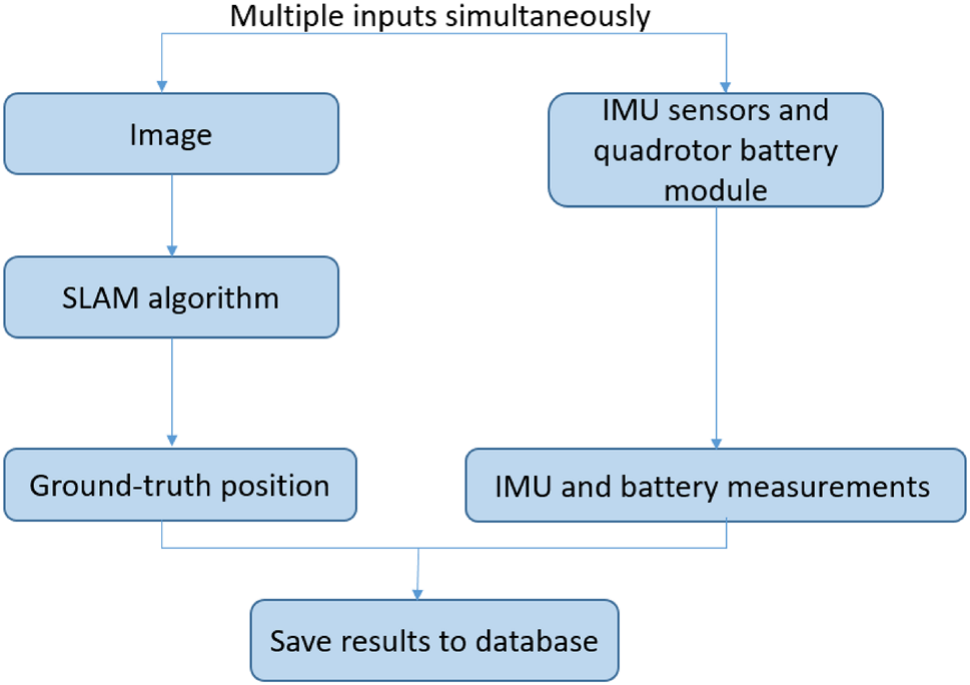
System architecture: two processes occur simultaneously. The first one uses the SLAM algorithm to output an accurate location. The second outputs IMU and battery measurements. The information is stored in a database.

Every experiment begins by stabilizing the quadrotor in a hovering state for a pre-defined time of three seconds. During the experiment, the Raspberry Pi records the outputs of the IMU sensor, battery, and SLAM algorithm, as described in Fig. 6. The quadrotor’s camera is used to process real-time images for the sole purpose of executing the SLAM algorithm. It returns a precise location and is considered our ground truth, including notifying us when the quadrotor has arrived at the intended experiment destination, which is set to be eight meters. It was confirmed that the laboratory had a sufficient amount of features for the SLAM system to recognize, along with appropriate lighting conditions resulting in a ground-truth accuracy of several centimeters. The battery usage was recorded to determine the battery consumption during the experiment. At the end of the experiment, the system stores in a database, for backup or retrieval purposes, all of the measurements that were taken. Additional data is stored in the database as meta-data, containing all the experiment’s details, such as the experiment name and time.

## Analysis and results

This section presents the evaluation outcomes of a series of experiments. Initially, the diverse scenarios of drive and flight are demonstrated, followed by an analysis of the trade-offs between distance error and battery consumption. The flight-only results are then presented, highlighting the various flying scenarios and the impact of utilizing the INS and QDR equations on distance error and battery consumption.

### Experiment scenarios

Our hybrid quadrotor has the ability to drive and fly. A natural question to ask is how its dynamics influence positioning accuracy in pure inertial situations and also what will be the resulting battery consumption. To that end, we examined seven different types of motions divided into two sets: 1) a mixture of driving and flying, and 2) only flying. The mixed driving and flying scenarios are:

1. Only driving.
2. 2.7 meters of flying followed by 5.3 meters of driving.
3. Four meters of flying followed by four meters of driving.
4. Two meters of flying, followed by two meters of driving, then another two meters of flying, and two meters of driving.

**Figure 7:**
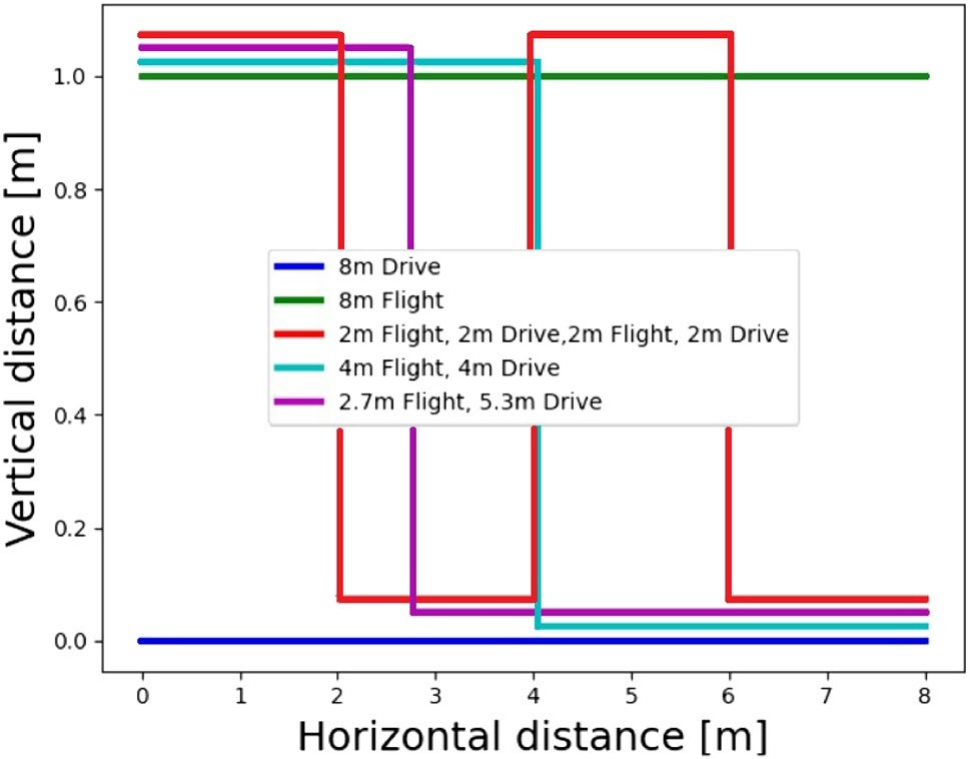
Illustration of the driving, flying, and a mixture of driving and flying trajectories.

The above set of trajectories is illustrated in Fig. 7. The second set includes the following flying scenarios:

1. Flying in a straight line.
2. Horizontal QDR (HQDR) periodic motion flight on the x-y plane.
3. Vertical QDR (VQDR) periodic motion flight on the z-x plane.

The VQDR and HQDR trajectories have a peak-to-peak period of 12 seconds and an amplitude of 20 centimeters resulting in 16 peaks during the trajectory. While all of the above trajectories length was set to eight meters and is considered the stopping criterion for the SLAM algorithm, the time duration is subject to variation due to several factors. These include the different amount of time the quadrotor executes an action, the message response time between hardware components, such as RPI and SLAM/DJI Tello, and the number of takeoffs and landings, which may vary between experiments, among other additional factors. To maintain a fair comparison between the different scenarios, the time duration was set to 100 seconds. In practice, the shortest experiment duration recorded was 96 seconds (in HQDR and VQDR experiments), while the longest one was 102 seconds. To evaluate the performance and compare the above seven scenarios, we use the absolute distance error (ADE), defined as:

20 $$\begin{aligned} {ADE}= & {} \mid {\varvec{d}}_{SLAM} - {\varvec{d}}_{IMU}\mid \end{aligned}$$

where $\varvec{d}_{SLAM}$ is the ground-truth distance of eight meters computed by the SLAM algorithm, and $\varvec{d}_{IMU}$ is the computed distance based on the inertial data.

### First order calibration

The first-order inertial calibration, described in “Inertial measurement unit (IMU): first order calibration” section, and its implementation procedure as addressed in “Experimental setup and design” section, is applied to the accelerometers and gyroscopes, and its influence on the distance estimation is evaluated. To that end, we employ an eight meter driving scenario and examine four different calibration options:

1. The accelerometers as well as the gyroscopes are calibrated (GAC).
2. Only the accelerometers are calibrated (AcC).
3. Only the gyroscopes are calibrated (GyC).
4. No calibration (NoC).

**Figure 8:**
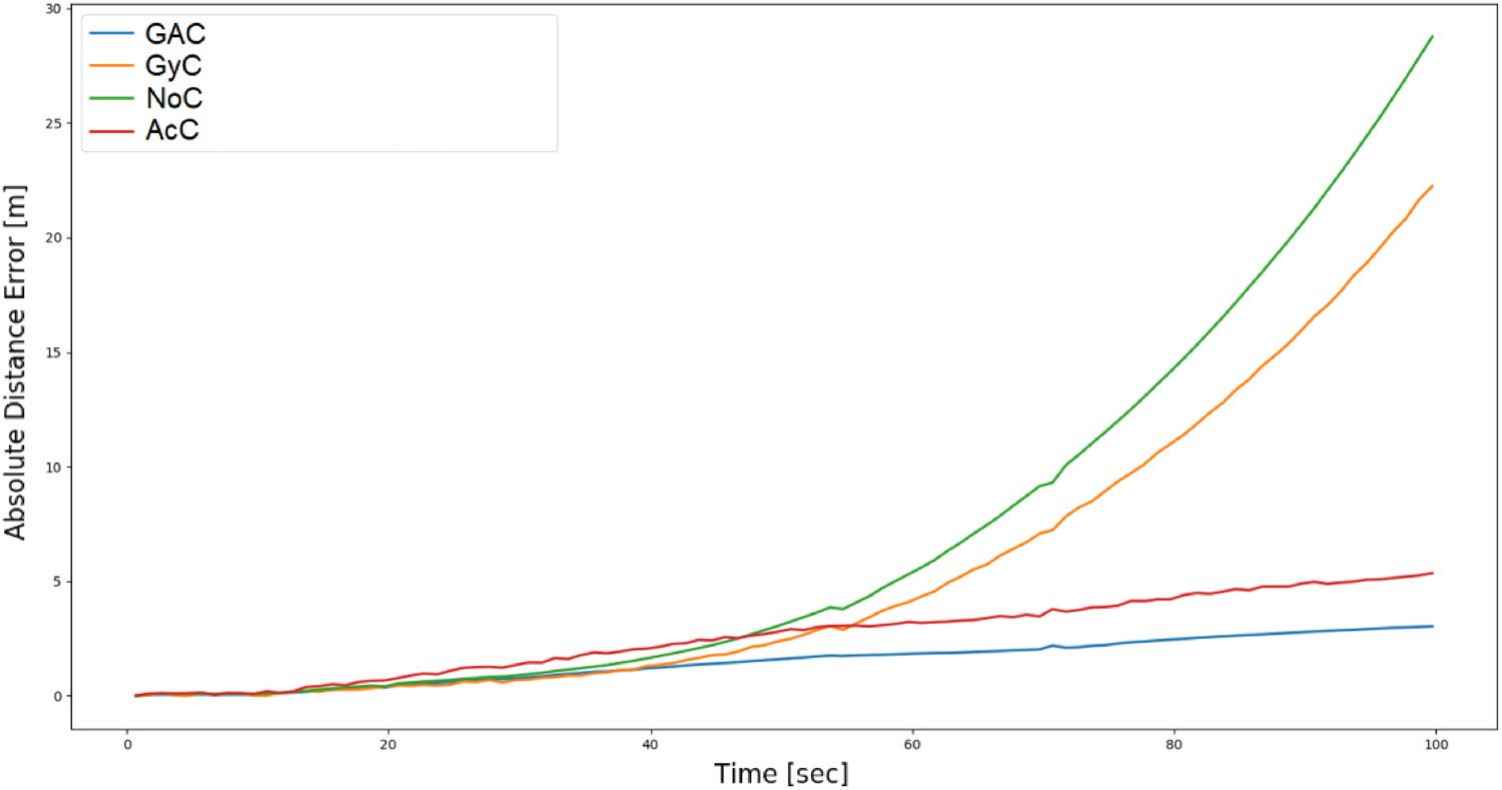
The influence of different calibration scenarios on the distance error for an eight meter long drive trajectory.

The estimated real-time position outputs are calculated using the INS equations (3)–(5). Given, the SLAM ground-truth solution and the INS solution we use (20) to calculate the ADE. The distance error of the above four scenarios is presented in Fig. 8.

In the absence of calibration (NoC), the highest error was obtained. Where, at the end of the trajectory the error is twenty-eight meters. The calibration of only the gyroscopes (GyC) managed to reduce the error to twenty-two meters, a 21.4% improvement. Accelerometer only calibration (AcC) better improved the performance by 82.1%. As a result, we observe that the influence of accelerometer calibration is more dominant. Finally, as expected, calibration of both accelerometers and gyroscopes (GAC) obtained the best performance with an error of three meters which corresponds to an improvement of 89.2% from the NoC scenario. Consequently, it becomes apparent that the calibration of both sets of sensors in the IMU is crucial to achieving minimal distance error.

### Driving/flying scenario results

To identify the various trade-offs associated with drive, flight, and a combination of both modes of transportation, a series of five experiments was conducted. Those comprise of the Set 1 trajectories (four mixed driving and flying scenarios) with an addition of a fifth scenario of only flying. Table 2 presents the ADE metric at the end of the trajectory, the battery consumption throughout the trajectory of each scenario, and the amount of time required to complete the eight meter trajectory.

**Table 2:** Driving/flying scenario results showing the ADE, battery consumption.

Scenario	ADE (m)	Battery consumption (%)
Drive—8 m	3	10
Fly—8 m	4	22
Fly—2.7 m, Drive 5.3 m	16	18
Fly—4 m, Drive 4 m	31	22
Fly 2 m, Drive 2 m, Fly 2 m, Drive 2 m	388	24

When solely driving, the distance error and battery consumption were found to be the lowest with an ADE of three meters and a battery consumption of 10%. Conversely, when only flying, the distance error was bigger by 33.3%, and the battery consumption was more than twice bigger reaching 22%. From a mission duration perspective, driving took one hundred and two seconds while flying took one hundred seconds, which is 2% faster.

When considering scenarios involving a combination of driving and flying, it was observed that the distance error was significantly larger compared to driving-only or flying-only scenarios, while the battery consumption closely resembled that of a flying-only scenario. For example, in the scenario of Fly—2.7 m, Drive 5.3 m, the ADE was increased by a factor of 5.3. The worst performance was obtained in the Fly 2 m, Drive 2 m, Fly 2 m, Drive 2 m scenario. This is attributed to the rapid change of dynamics influencing the inertial measurements.

From a trade-off perspective, it can be argued that driving offers the optimal choice for minimizing both distance error and battery consumption. However, in situations where the destination cannot be reached solely through driving or where time constraints exceed the capabilities of drive navigation, the utilization of flying capabilities becomes necessary.

### Flying scenario results

In this section, we are focused on our Set 2 of scenarios which contain three different flying scenarios. For each scenario, five experiments were made. Table 3 presents the ADE metric at the end of the trajectory, the battery consumption throughout the trajectory of each scenario, and the amount of time required to complete the eight meter trajectory. As fifteen experiments were made, we also present the corresponding standard deviation (STD). Notice that for both QDR scenarios (HQDR and VQDR) we calculated both the regular INS solution based on (3)–(5) and the QDR solution using (14)–(16).

**Table 3:** Flying scenario results showing the ADE, battery consumption.

Scenario	ADE (m)	Battery consumption (%)	Distance error STD (m)	Battery STD (%)
Fly—8 m (INS)	4	22	2.85	3.16
HQDR—Fly 8 m (QDR)	0.96	26.6	1.29	4.28
HQDR—Fly 8 m (INS)	6	26.6	3.27	4.28
VQDR—Fly 8 m (QDR)	0.87	27	1.03	3.57
VQDR—Fly 8 m (INS)	16	27	5.11	3.57

The results show that through the QDR approach, in both HQDR and VQDR scenarios, a considerably smaller mean distance error is obtained compared to a straight flying scenario. Both HQDR and VQDR improved by more than 75% the straight flying scenario, where VQDR obtained an improvement of 78.25%. On the other hand, as expected, the battery consumption of QDR trajectories is over 20% higher than that of straight-flight motion. Moreover, comparing the results to the driving scenario, VQDR reduces the ADE to 0.87 meters resulting in a 71% improvement but with a cost of 170% more of battery consumption.

### Results summary

**Figure 9:**
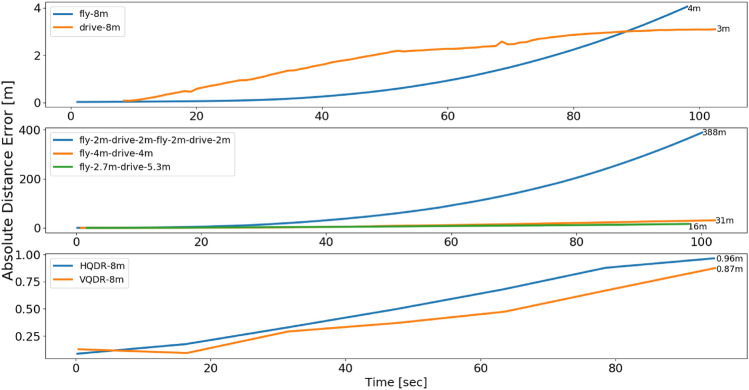
ADE as a function of time for the examined seven scenarios.

**Figure 10:**
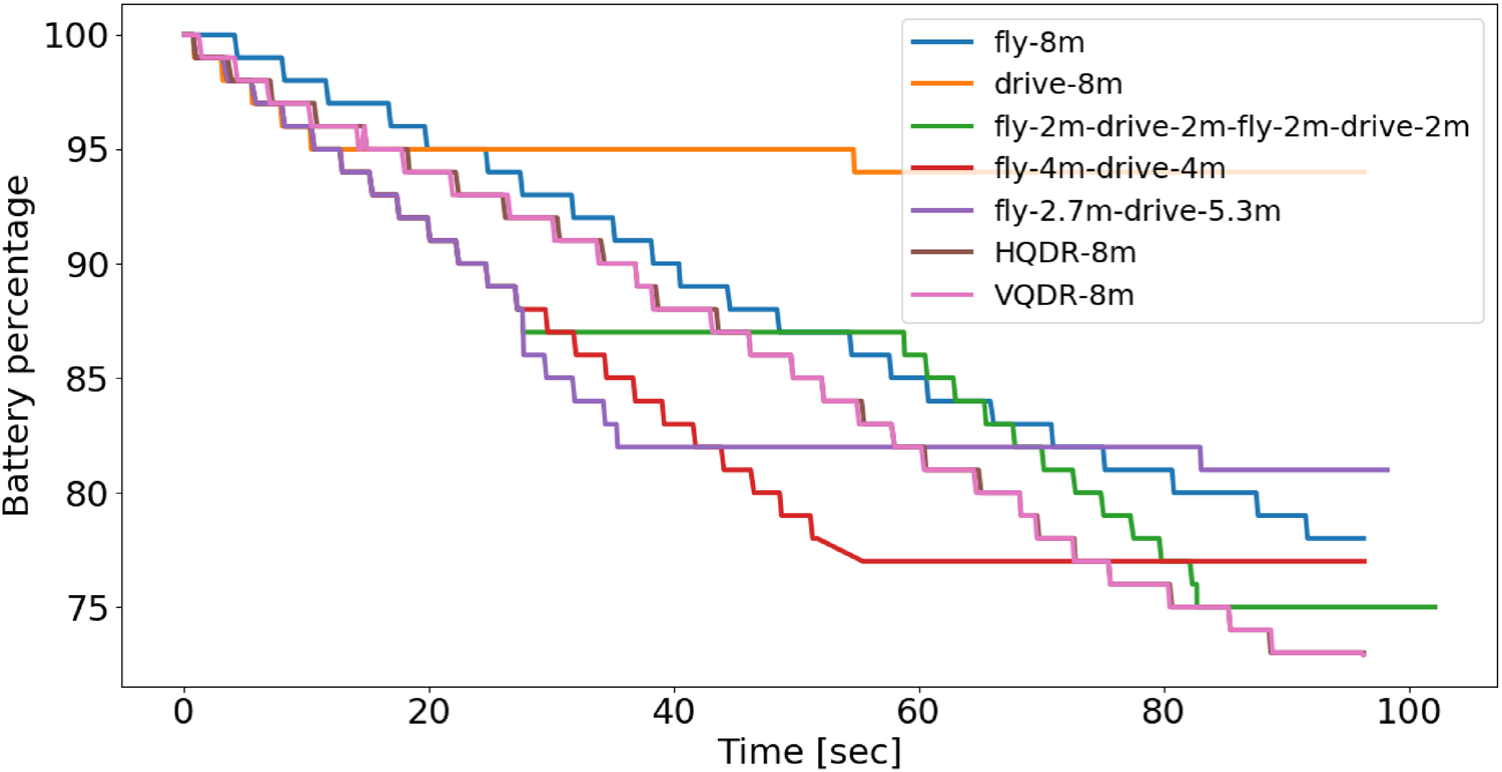
Battery percentage as a function of time for the examined seven scenarios.

The ADE as a function of time during all different scenarios (Set 1 and Set 2) are presented in Fig. 9, and the battery percentage in Fig. 10. The battery consumption was as low as 1.25%/m for only driving and increased to 2.75%/m for only flying, and 3.37%/m for VQDR. In terms of the ADE metric, VQDR obtained the best performance.

## Conclusions and future work

This paper introduces a novel and cost-effective wheeled quadrotor configuration that was designed with hybrid abilities allowing both driving and flying capabilities. Our design allowed the driving capability with an increased weight of only fifteen grams, resulting in less than 20% of the added weight.

The configuration incorporates a comprehensive array of sensors, including accelerometers, gyroscopes, and a camera. With the added driving option, many different flying/driving possibilities now exist. Each one leads to a different positioning accuracy and battery consumption. To examine and asses those possibilities, we defined seven different trajectories with only driving, only flying, and mixed driving/flying scenarios. All trajectories were eight meters long.

Our results show that when only driving the battery consumption was the lowest with 10%. On the other hand, VQDR reached the minimum ADE of 0.87 meters improving the only driving scenario ADE by 71%. This comes with a cost of an additional 170% of battery consumption.

A combination of driving and flying can be used when necessary based on environmental constraints, but may lead to significantly larger errors. Our results show that a trade off exists between positioning accuracy and battery consumption. If minimizing distance error is a top priority, applying a periodic motion strategy during flight to reach the destination should be the primary consideration. However, this comes at a cost of high battery consumption. On the other hand, if battery consumption should be minimized, driving the quadrotor on the ground is the most efficient option.

Additionally, our results demonstrate that the distance error and battery consumption are significantly low during driving mode, highlighting the practicality of our proposed configuration in various real-world scenarios. Our future research will involve assessing additional scenarios focusing on mission planning optimization to achieve the optimal balance between flight and drive time while ensuring precise localization of the quadrotor. As well, we plan to upgrade our design to cope with possible quadrotor crashes and uncontrolled quick landings that may damage or break the wheels.

Our proposed wheeled quadrotor has demonstrated that driving capabilities can extend the mission duration as less energy is required. When designing for a specific mission, it is possible to adjust the weight distribution between components and the battery to meet the requirements. For instance, in scenarios where driving is feasible, such as indoor deliveries or search missions, integrating a driving chassis could offer a superior, cost-effective, and more dependable solution, resulting in a smaller drone than one without driving capabilities. Taking into account all the essential components, a hybrid system may prove to be a more economical alternative to a purely flying one.

## Data Availability

Correspondence and requests for materials should be addressed to D.F. or I.K.
